# Views of Canadian patients on or nearing dialysis and their caregivers: a thematic analysis

**DOI:** 10.1186/2054-3581-1-4

**Published:** 2014-04-07

**Authors:** Lianne Barnieh, Kathryn King-Shier, Brenda Hemmelgarn, Andreas Laupacis, Liam Manns, Braden Manns

**Affiliations:** Department of Medicine, University of Calgary, Calgary, Alberta Canada; Interdisciplinary Chronic Disease Collaboration, Kragujevac, Alberta Canada; Department of Community Health Sciences, University of Calgary, Calgary, Canada; Libin Cardiovascular Institute and Institute of Public Health, University of Calgary, Calgary, Canada; Faculty of Nursing, University of Calgary, Calgary, Alberta Canada; Keenan Research Centre, Li-Ka Shing Knowledge Institute of St. Michael’s Hospital, Toronto, Ontario Canada; Faculty of Medicine, University of Toronto, Kragujevac, Ontario Canada; Foothills Medical Centre, 1403 29th St., NW, Calgary, Alberta T2N 2T9 Canada

**Keywords:** Content analysis, Kidney disease, Needs

## Abstract

**Background:**

Quality of life of patients receiving dialysis has been rated as poor.

**Objective:**

To synthesize the views of Canadian patients on or nearing dialysis, and those who care for them.

**Design:**

Secondary analysis of a survey, distributed through dialysis centres, social media and the Kidney Foundation of Canada.

**Setting:**

Pan-Canadian convenience sample.

**Participants:**

Patients, their caregivers and health-care providers.

**Measurements:**

Text responses to open-ended questions on topics relevant to end-stage renal disease.

**Methods:**

Statements related to needs, beliefs or feelings were identified, and were analysed by thematic content analysis.

**Results:**

A total of 544 relevant statements from 189 respondents were included for the thematic content analysis. Four descriptive themes were identified through the content analysis: gaining knowledge, maintaining quality of life, sustaining psychosocial wellbeing and ensuring appropriate care. Respondents primarily identified a need for more information, better communication, increased psychosocial and financial support for patients and their families and a strong desire to maintain their previous lifestyle.

**Limitations:**

Convenience sample; questions were originally asked with a different intent (to identify patient-important research issues).

**Conclusions:**

Patients on or nearing dialysis and their caregivers identified four major themes, gaining knowledge, maintaining quality of life, sustaining psychosocial wellbeing and ensuring appropriate care, several of which could be addressed by the health care system without requiring significant resources. These include the development of patient materials and resources, or sharing of existing resources across Canadian renal programs, along with adopting better communication strategies. Other concerns, such as the need for increased psychosocial and financial support, require consideration by health care funders.

## Abstract (fr)

**Contexte:**

On décrit souvent la qualité de vie des patients en dialyse comme mauvaise.

**Objectif:**

L’objectif de cette étude est de présenter les perspectives de patients canadiens en pré ou en dialyse ainsi que celles de leurs proches.

**Type d’étude:**

Analyse secondaire d’un sondage effectué via les centres de dialyse, les médias sociaux et la Fondation canadienne du rein.

**Échantillon:**

Échantillonnage de convenance pancanadien.

**Participants:**

Les patients, leurs aidants naturels et les professionnels de la santé.

**Mesures:**

Réponses aux questions ouvertes relatives à la maladie rénale terminale.

**Méthodes:**

Les affirmations relatives aux besoins, croyances et sentiments ont été identifiées et une analyse thématique et de contenu a été effectuée.

**Résultats:**

Nous avons inclus 544 affirmations pertinentes au sujet de recherche provenant de 189 répondants dans notre analyse de contenu. Nous avons identifié quatre thèmes descriptifs lors de l’analyse de contenu : l’acquisition des connaissances, le maintien de la qualité de vie ainsi que du bien-être psychosocial et des soins appropriés. Les répondants ont mentionné un besoin d’avoir plus d’informations, une meilleure communication, un support psychosocial et financier accru pour les patients et leurs familles et un désir intense de maintenir leur style de vie antérieur à la maladie.

**Limites de l’étude:**

L’échantillon de convenance ainsi que le fait que les questions avaient une visée différente (identifier les domaines de recherche importants pour les patients) constituent des limites de cette étude.

**Conclusion:**

Les patients en pré-dialyse et en dialyse ainsi que leurs aidants naturels ont identifié 4 thèmes majeurs : l’acquisition des connaissances, le maintien de la qualité de vie ainsi que du bien-être psychosocial et l’accès à des soins appropriés. Le système de santé pourrait se pencher sur certains de ces thèmes sans avoir recours nécessairement à des ressources supplémentaires. Ceci pourrait se traduire par le développement de ressources pour les patients ou le partage de ressources existantes entre les programmes canadiens de néphrologie ainsi que le développement de meilleures stratégies de communication. Les autres préoccupations identifiées lors dette recherche, telles qu’un meilleur support financier et psychosocial, doivent être cependant examinées par les organismes responsables du financement en santé.

## Background

End-stage renal disease (ESRD) is increasing worldwide; at the end of 2009 there were nearly 38,000 people living with end-stage renal disease in Canada [1]. Most patients with ESRD do not receive a kidney transplant and require dialysis to survive. Quality of life for patients receiving dialysis has been rated similarly to patients with metastatic cancer [2], in part because of the intrusiveness of ongoing dialysis (whether hemodialysis or peritoneal dialysis), and in part because of symptoms related to the disease, including depression which occurs in up to 30% of patients [3, 4].

Qualitative research provides insight into the views of respondents, including their attitudes, needs, beliefs and feelings. This provides some insight into patients’ perspectives on their illness. Understanding patients’ views can help health care providers tailor the type of treatments offered to patients, as well as understand when, how and what information to provide to patients about the treatment options. This is particularly relevant in patients with chronic diseases, such as kidney failure, since they deal with their illness and the consequences of its treatment on a daily basis.

We build on prior work to determine the research priorities of patients on or nearing dialysis, their caregivers and the health care professionals who look after them. To develop the list of top research priorities, we initially surveyed patients, their caregivers and health care professionals across Canada, collating, and ranking their responses. A top ten list was subsequently developed at an in-person meeting [5]. During this priority setting exercise, we noted that many of the items expressed by respondents represented not only unanswered research questions, but also expressed the needs, beliefs or feelings that were specifically related to their illness or its treatment. In this report, we use survey responses from patients and caregivers to conduct a thematic analysis of their views. This consideration of the views of patients and caregivers with kidney disease is meant to inform health care service providers about ways to support treatment decision-making, enhance communication, address psychosocial wellbeing and improve patient satisfaction.

## Methods

We undertook a qualitative, descriptive study, based on a secondary analysis of survey data, using thematic content analysis to synthesize the views of patients on or nearing dialysis and their caregivers.

### Participants

Patients, caregivers and health care professionals who care for patients on or nearing dialysis were invited to complete a survey distributed online through partner organizations (Kidney Foundation of Canada), social media (Twitter, Facebook), or a paper-based version available in 10 Canadian hemodialysis centers. The survey was open for 3 months (October 2012 – January 2013). For this analysis, we only analyzed the responses of patients and their caregivers.

### Survey

The survey consisted of seven open-ended questions on topics relevant to patients with ESRD (overall management of kidney failure, treatment options, dialysis access options, prognosis, diet, symptoms and lifestyle). These open-ended questions were intended to elicit questions that were answerable by research, for example: “Are there questions about decisions regarding the way in which kidney failure can be treated that you would like to see answered by research?” In previous work, these research questions were prioritized to develop the top ten most important unanswered research questions from the perspective of patients and their caregivers (http://www.cann-net.ca/patient-information/dialysis-research-priorities-survey#results). While the goal of this previous work was to consider respondent statements in the context of developing a list of unanswered research questions, in the current work, we were interested in how the statements reflected respondents, needs, beliefs and feelings. In the survey responses, some patient statements only reflected unanswered research questions, and did not inform views. To eliminate these statements, which were less informative in the context of this work, two reviewers classified all responses independently, and separated these statements out. The remainder of the statements formed the dataset for the content analysis.

### Qualitative content analysis

Statements were analyzed by thematic content analysis, a method that enables systematic analysis of the content in communication, such as answers to a survey, and reflects the content of the data set. Thematic content analysis is a method for identifying, analyzing, and reporting themes within data, where concepts or categories are derived from the data in an inductive or deductive manner [6]. These concepts or categories serve to represent the data by providing knowledge and new insights, with the outcome a condensed and broad description of the phenomenon under study [7]. Content analysis goes beyond the summary of the data and involves a qualitative interpretation by the researcher, providing a “vicarious experience” for the audience [8].

A descriptive theme was defined as a subject that captured important data related to end-stage renal disease[9]. Themes were not defined a priori but emerged after the initial readings of the data. Themes were identified as having captured an important element of the views of patients with chronic kidney disease and their caregivers and were not necessarily the most prevalent subjects within the data. Themes were not created to be mutually exclusive, but in order to represent the best conception of the data.

### Synthesis of findings

We followed the process outlined by Braun et al. [6] to synthesize the results. The data were first read by one author (LB) several times, to become familiar with the content and generate an initial idea of themes. The thematic content analysis involved three phases: the development of descriptive themes along with the creation of mutually exclusive rules for inclusion; statement by statement coding done independently by two authors (LB & LM); and the organization of the statements into meaningful categories by the same two authors (LB & LM). Any disagreements between the two authors were resolved by consensus, with the input of a third reviewer (BM) if necessary. We defined 4 descriptive themes, which were re-assessed to ensure that they worked with and represented the data. Lastly, a selection of powerful and compelling examples were selected, with context, to represent each theme.

## Results

The survey elicited a total of 1820 statements from all respondents (patients, health care providers and caregivers) (Table 1). Of these, 245 were removed because they were out of scope (not relevant to patients on or nearing dialysis) or were unclear. Of the remaining 1575, 1054 were from patients or caregivers. Statements that only expressed a research question, for example “can stem cell research improve my kidneys” or “what research is being done with regard to a mechanical replacement kidney, much like the artificial heart”, were removed. There were a total of 544 remaining statements, from 189 individual respondents, included for the thematic content analysis.

**Table 1:** **Patient and caregiver characteristics**

Type of respondent	n (%)
	Total n= 189
Patient receiving hemodialysis in a clinic	91 (48.1)
Patient on home hemodialysis	32 (16.9)
Patient on peritoneal dialysis	22 (11.6)
Patient on dialysis, no detail	2 (1.1)
Patient, within a year of starting dialysis	6 (3.2)
Care provider	36 (19.0)
**Age**	
18 – 29	5 (2.6)
30 – 39	15 (7.9)
40 – 49	26 (13.8)
50 – 59	35 (18.5)
60 – 69	49 (25.9)
70 – 79	23 (12.2)
80 and over	10 (5.3)
Prefer not to answer	26 (13.8)
**Gender**	
Male	73 (38.6)
Female	89 (47.1)
Prefer not to answer	27 (14.3)
**Ethnicity**	
Aboriginal	3 (1.6)
Asian	10 (5.3)
Black	9 (4.8)
Mixed	3 (1.6)
Other	7 (3.7)
White	123 (65.1)
Prefer not to say	34 (18.0)
**Province**	
Atlantic	26 (13.8)
British Columbia	7 (3.7)
Ontario	59 (31.2)
Prairies	65 (34.4)
Quebec	4 (2.1)
Territories	1 (0.5)
Prefer not to say	27 (14.3)

**Figure 1:**
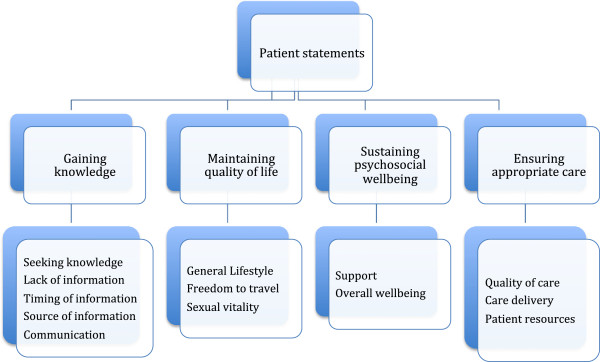
**Components of each theme as an overview of the thematic analysis.**

Four major descriptive themes were identified as being central to the views of patients with chronic kidney disease and their caregivers: gaining knowledge, maintaining quality of life, sustaining psychosocial wellbeing, and ensuring appropriate care. A thematic schema of the analytical framework is represented in Figure 1.

## Gaining knowledge

The theme gaining knowledge encompassed seeking knowledge/lack of information, communication, timing of information, and included how patients and their caregivers learned about topics related to their chronic kidney disease.

### Seeking knowledge/lack of information

Respondents sought knowledge not only about the different treatment options available (either dialysis modality or access), but also wanted to determine which was the “best”, and why, for both their overall health and quality of life. They specifically expressed the desire for a thorough explanation of all options. A caregiver stated: “*Yes, I would like to see all of these options thoroughly explained at the very beginning. This is a whole new world for patients and care givers, making it very difficult to make good decisions*.” Respondents verbalized that they felt uninformed about many aspects of their treatment and care. A hemodialysis patient expressed: “*Again left in dark till things happened, policy seems to be on a need to know. When you start dialysis why don't they warn you about the symptoms of crashing? Not nice not knowing what is happening”.* There was a recurring concern regarding symptom management, and respondents frequently voiced that they were unsure of why certain complications occurred (lack of energy, itchiness). They also felt that the lack of information interfered with their ability to cope with and manage these symptoms, as stated by a hemodialysis patient: “*Why do we get restless legs, itchy skin and have difficulty sleeping? No one ever explained why nor what can be done*”. This lack of information extended to other areas of their disease, including fluid management and diet restrictions. A peritoneal dialysis patient wrote: *“More information on why some foods are okay and others aren't. I had to find detailed lists of phosphate containing foods on the internet*”.

Respondents also stated that they were unsure about their prognosis on dialysis, wanting to know how long they would need to stay on dialysis, the progression of their disease, as well as their life expectancy. A patient receiving hemodialysis in a clinic expressed: “*Generally a summation of what will happen physically as the years pass on dialysis would be beneficial to me as a patient. What should I expect in terms of medical problems and deterioration due to the end stage renal disease*”. Lack of information extended to those seeking a transplant as well. A patient receiving hemodialysis in a clinic wanted to know: “*At what age, and with what present medical issues does a person with kidney failure become ineligible for a transplant? Persons with kidney failure want to know all about the process of selection regarding a transplant*”. One dialysis respondent expressed that they wanted a holistic take on information, stating *“We need to be better informed about the side effects of dialysis on your mind as well as your body, such as focus, cognitive ability and lack thereof. Also, how your whole life will change: how you have to live and interact with others and how totally frustrating it can be”.*

### Communication

In addition to not receiving the information they felt they needed, participants raised concerns about the communication between patient and healthcare provider, specifically in how the information was delivered. “*An explanation in layman's terms would be useful*”. A hemodialysis patient felt information was not always communicated appropriately. “*I started dialysis two weeks ago. There are a number of acronyms used, which I do not understand. How can we improve communication with patients?*” This same respondent expressed that he found it “*very frightening*” when first discussing kidney disease.

Respondents also expressed a desire for improved communication for managing their disease, and being informed. A home hemodialysis patient wondered: “*Why can’t the nurses share what’s going on with our blood work and inform us as patients*”. In the particular case of diet restrictions, a long term dialysis patient stated: “*Just because the patient was told this once at the beginning does not mean they remember. There should be some kind of annual review of the do’s and don’ts as well as product updates*”. A respondent receiving hemodialysis in a clinic stated it as follows: “*They need to go one step further than just give us a piece of paper saying what you can and cannot eat or drink and explain the consequences of not following a diet and watching what you drink*”.

Many respondents verbalized their desire for a transplant, but felt that the selection process and position on the waiting list could be communicated in a clearer manner. Some felt this process was not transparent, perhaps due to a lack of communication on how the process works. “*Why do I have to wait so long? Why haven't they made an effort to get me on the transplant list? Will I ever get my transplant?*”.

Overall, some respondents voiced that they would like to feel part of the team and more involved in the decision making around their care. This was expressed by a patient receiving hemodialysis in a clinic through the following statement: “*I’ve been through being diagnosed and I still see it happening: the nephrologist tells you what you have, the treatment and the diet, but never acts like you’re a part of the team. Why is it that up until the moment you are told the above, you think you have a say in your treatment, but the specialist doesn’t see it that way*”.

### Timing of information

Respondents also questioned whether information could have been given earlier in the disease process. They felt that had they known the severity of their disease earlier on, they could have taken more action to prevent or delay the progression. Respondents also wanted to know early on about their treatment options for dialysis, given the impact of the decision. One patient wrote, “*I feel that the patient should be told earlier about their choices. Also important knowing about how dialysis can affect your life and that of your family, friends, employer, etc. It is very traumatic and life changing*”.

For those who had already made a decision about dialysis options, a respondent receiving hemodialysis in a clinic recounted feeling pressure to make a decision and needed more time. “*Can you make the whole orientation process much clearer? Also, a strong focus was given to home hemodialysis, which I wasn't comfortable doing. Pushing us, especially those on the senior side, to use home dialysis with that machine is terrifying, even with training. Can you build layers of consulting to ease those of us into our new lifestyle? It may be repetitive but we'd make better decisions in the long run*”. Another patient receiving hemodialysis in a clinic recounted his experience as follows: “*When I first started dialysis, it would have been very helpful to know about the side effects of dialysis. I believe it would have helped prepare myself and my spouse for the onset of all this stuff. I received very little information when I first started*”.

### Source of information

Peers appeared to be an important influence for both patients and caregivers on deciding their treatment options. Respondents valued the experience of their peers in the process of choosing their modality and were interested to know how they came to their decision. One caregiver stated: “*Indeed it was a very difficult decision to make regarding the topic of which dialysis treatment would be best suited for us. We could have used more info on real people who have done both and what their experiences were and what the pros and cons are. Also the success rate of each and is there any difference or research on who does better on the different treatments”.* Respondents also looked to their peers in terms of their care, benchmarking their treatment against others, and wanting to know why there were differences. “*How come some people have shorter times [on dialysis] than others, and more frequently during the week?*”.

## Maintaining quality of life

The theme maintaining quality of life encompassed the patient’s general lifestyle, freedom to travel and sexual vitality. These were all identified as being important in maintaining a quality of life similar to pre-dialysis.

### General lifestyle

Respondents most often expressed a strong desire to maintain their lifestyle, prior to the commencement of dialysis, what they commonly referred to as a “*normal life*”. One respondent receiving hemodialysis in a clinic expressed: “*What are the best options for a normal life*?”. Respondents also wanted to know how they could plan a life and maintain work while undergoing dialysis, as one respondent phrased it: “*How to plan a life and work style that is compatible with staying fit while undergoing dialysis”*. One dialysis patient expressed a desire for normalcy and control in her ability to prepare the appropriate foods: *“I see cookbooks for people of all kinds of conditions - heart problems, diabetes, gluten intolerance etc. I have never come across a cookbook for kidney patients. Can't some dietitian make one for us?”.* Respondents who experienced side effects or complications from dialysis or their illness often described the impact this had on their quality of life. “*I've lost my eyesight and leg because of diabetes-how can I have a normal life? My wife is fed up, tired of my problems, how can I make this better? I need her to look after me*”.

### Freedom to travel

A large proportion of respondents verbalized their desire for freedom to travel and stated that traveling was extremely important in maintaining their quality of life. Respondents identified several barriers related to travel. Limited access to other dialysis centers was one barrier identified by a respondent on hemodialysis: “*Can more access to hemodialysis machines be set up so that people who like to travel can get appointments in different parts of the country? It is very difficult to get time to dialyze where we boat and like to camp*”. Another respondent identified not knowing how to plan travel while on dialysis and resources available for travel (both personal resources required and those made available by the health system) as barriers. “*Can persons on dialysis be provided with a comprehensive and detailed directory regarding travel anywhere throughout Canada, including any special perks or considerations available by public or private services in terms of costs, access, assistance, etc.”.* Respondents were also concerned with whether it was safe to travel while dialyzing and any possible consequences traveling had on their health.

### Sexual vitality

Respondents expressed the desire for more forthright communication around sexual vitality, emphasizing that sexuality was part of maintaining a normal life for them. A young woman with kidney disease expressed it as follows: “*Sexuality is never spoken about. Young patients often have many questions about fertility, sex and how to deal with having a line or catheter and a sexual life*”. Respondents, and family members, were especially concerned about how they could maintain their sexuality, either by addressing erectile dysfunction, lack of sexual desire or other challenges to sexuality related to dialysis itself (e.g. femoral dialysis lines). “*People are complaining about groin lines and trying to understand how they can still have sex. Sex is very important to any couple. Open communication regarding this topic would be much appreciated. All we need is to be on dialysis and divorced!”.*

## Sustaining psychosocial wellbeing

The theme sustaining psychosocial wellbeing included psychosocial support for both patients on or nearing dialysis and their caregivers, in addition to their overall wellbeing.

### Support for themselves & caregivers

Respondents were concerned about their access to support for themselves and their caregivers, whether at the start of treatment or after years on dialysis. “*How about having a counseling session of encouragement before a patient goes on dialysis because I was terribly afraid and I wanted to run away*”. One caregiver expressed a need for psychosocial care. “*What do you do about the mental care of a dialysis patient that suffers from depression due to or caused by lengthy treatment of dialysis?*”. Respondents were also concerned about their caregivers and the toll the disease took on them, illustrated by the following two quotes from patients on hemodialysis. “*What types of things do family members have concerns about but are not voicing? What effect does CKD have on them*?”. “*My wife left me because I was no longer that strong man she married, she wasn't getting the emotional love she needed and she thought I was going to die. Can more support/documents be made available to the spouses?”.* Some described the need for psychosocial support that extended beyond the dialysis unit. “*Someone outside of the dialysis unit that is familiar with personal issues of chronic kidney disease other than my family doctor, especially dealing with sleeping and sexuality*”.

### Overall wellbeing

Respondents emphasized that the lack of quality of life they experienced while on dialysis contributed to feelings of depression or inadequacy. “*I cannot make myself breakfast/get dressed/put on socks. My quality of life is very bad but I feel all these symptoms are a part of my life. All I can do is smile and keep doing what I can, but it is very hard*”. Many comments reflected the overwhelming and deep-seated impact of dialysis and kidney disease on everyday life. “*Without work you are living under the poverty line so are not only dealing with loss of health but now can barely afford to live. Family wants to distance themselves so they won't be reminded that death is imminent and won't feel the pain. Canada has made travel impossible. Most dialysis places don't want dialysis patients from another place, for various reasons; might infect their patients, too much trouble to accommodate. We cannot leave the country unless we are willing to pay for our own dialysis care. We are already destitute financially so that is impossible unless you have money. These kinds of restrictions, along with diet, and overall health add to feelings of depression”.*

## Ensuring appropriate care

The theme ensuring appropriate care included quality of care, care delivery and patient resources. Quality of care encompassed the satisfaction and standard of care patients received, while care delivery centered around the health system and its resources that impacted care of the patient. Patient resources focused on any perceived or real limitations to accessing health care due to constraints of personal financial resources of patients.

### Quality of care

Few respondents communicated concerns about their quality of care, with many expressing satisfaction with their nephrologists and other members of their health care team. “*No questions but very satisfied with all treatment and information by all dialysis staff I have had contact with*”. Respondents did question whether their disease could have been diagnosed earlier, and suggested that education of their primary care physician may have led them to being treated earlier. “*How do we educate family doctors to test for and refer patients at an early stage, so it is not such a shock when they are told they have kidney failure*”.

### Care delivery

Many of the views on care delivery expressed by respondents centered around perceived or real constraints on resources that affected their access to care, due to resources at the level of the health system. For example, some respondents perceived that home hemodialysis was a better treatment option, and that this should be offered more. “*Why are more people not trained and guided towards self care, peritoneal dialysis or home hemodialysis*”. Another example was the lack of availability of all treatment options at all health centres, as stated by one patient receiving hemodialysis in a clinic. “*I would like to try the button hole method but because of time and money and the clinic’s part, I have not been able to try it. I’m trying to find a way to preserve the life of my vein*”.

### Patient resources

As respondents are often limited in their ability to work and earn income, they were concerned about personal costs incurred because of their illness and the need for dialysis, as well as whether one dialysis therapy would incur more expenses to them: “*What personal costs could I expect if I chose one [dialysis therapy] over the other?*” Several dialysis patients expressed concerns about the impact that medication costs have on their finances, as highlighted by the following two comments. “*We need certain medications to deal with kidney failure and dialysis, so why aren't they free?*” “*Why are some medications covered and others not, for example sensipar? Last year I spent over $20,000 on medications. How does a 18-year dialysis patient survive his co-pay while maintaining their dignity and live with these costs?”* Another hemodialysis patient expressed frustration about the lack of financial resources available to them. “*Since dialysis patients can't work full-time because it takes time out of your life, why aren't we compensated financially? We need dialysis to survive*!”

## Discussion

We identified four themes that emerged from views of patients’ on or nearing dialysis and their caregivers: gaining knowledge, maintaining quality of life, sustaining psychosocial wellbeing, and ensuring appropriate care. Respondents identified a lack of information and communication between patient and provider as a significant concern, particularly with respect to information about dialysis modality and access. Respondents also expressed significant concerns about their quality of life on dialysis, and expressed a desire to have as much of a “normal” life as possible. They identified a variety of issues that could be addressed by both the health system and health care providers to improve their quality of life. With respect to the health system, there is an urgent need for more psychosocial support, and resources to facilitate travel, particularly for patients on hemodialysis. For health care providers, better communication is key, along with more information on why symptoms happen, how they can best be managed, and information about their future prognosis.

The majority of respondent’s expressed a need for gaining knowledge, which was thought of either as seeking knowledge or lack of information. This need for information was expressed by both dialysis patients and caregivers, echoing previous studies that found that the primary need of families of chronic dialysis patients was information [10, 11]. The timing of when information was presented to patients and caregivers was also a common theme, as was the importance of re-enforcing and updating material previously communicated. The information needs of people as they pass through the various stages of their disease changes over time. A careful consideration of the type of information, including how much information, and at what stage of their illness it is provided, could enable a more efficient transfer of knowledge from health provider to patient. Further, education may alleviate concerns and stress, and stress has been found to affect dialysis modality selection [12]. Providing predialysis education can not only enable patients to choose the modality best suited to them, but helped in their understanding of their disease[13], and may extend their time prior to dialysis initiation [14]. Indeed, previous research has noted that patients need information spread over an extended period of time, with increasing amount of detail and specificity as renal replacement therapy nears [15]. Clinical practice guidelines in the UK specify that information should also be tailored to the stage of disease [16].

Our results also highlighted that transferring knowledge is dependent on the quality of communication among people, echoing the results of a study that found that perceived knowledge of kidney disease was related to the quality of communication [17]. Many respondent comments reflected the need for improved communication between patients/caregivers and health care providers, though increased communication is not merely repetition of the same information. Improving health literacy of patients with chronic kidney disease may be one step in not only ameliorating communication, but may also addressing the information needs of these patients, and how knowledge is retained [18]. Health literacy encompasses communication between patients, their social networks, and providers. Limited literacy has been linked with reduced knowledge, less adherence, hospitalization and death [19, 20]. Finally, many respondents identified the importance of peer influence as a source of information, which may be particularly important since previous studies identified peer support as a means of providing practical information about kidney disease [21]. Peer support may also give purpose to patients already on dialysis by valuing their experiences. As peers have “been there, done that”, they have first hand comprehension of some of the difficulties of treatment, and may be a more influential source of information than clinicians for some important treatment decisions.

We identified the need for more resources for patients and their caregivers, including improved access to travel, more information on how to cook meals within the limitations of the renal diet, more financial support, and more psychosocial care for themselves and their caregivers. Patients on hemodialysis consistently requested more information on how to arrange hemodialysis in other cities, and were concerned with the lack of hemodialysis spots available to enable travel. With respect to meal planning, although there is high-quality information available online to assist dialysis patients and caregivers (http://www.kidneycommunitykitchen.ca/kkcookbook), this information may not be sufficiently broad to address all cultures, tastes or financial means. Further, the number of comments we received requesting additional meal planning resources suggests patients are not aware of existing resources and are still facing barriers with respect to meal preparation. The difficulty of preparing meals to meet the dietary restrictions of those on dialysis is a need that has been met by pre-packaged frozen meals in the United States [22]. Finally, with respect to the need for psychosocial care, the availability of trained professionals for psychosocial care in and out of the dialysis unit, along with support for caregivers, may help relieve the burden of living with this disease. Moreover, since depression is very common [23], resources to help patients prevent and manage depression appears to be a critical need for patients. In addition to professional support, social support has also been found to improve outcomes, including compliance, in those with end-stage renal disease [24, 25] and resources should be made available to help foster important social relationships.

In light of this information, kidney care and dialysis programs, in collaboration with patients and caregivers, should take inventory of what information they provide, the resources available, and how this information is communicated to patients. A careful examination of where a program’s current resources are being directed and whether this aligns with the needs of patients and their caregivers is necessary. Further, detailed information on how to access resources should be made clear and reiterated often. Although the challenges that patients face may vary slightly across the country, there are likely more similarities than differences in many of the views of patients. As such, the development of certain educational resources, including materials and access to online resources, could be a collaborative process across programs, reducing the need for duplication of effort. While these educational materials will not replace face-to-face visits, they may provide helpful information for patients needing further information in between visits. Programs may also explore the idea of shared decision making as an innovative way of communicating and involving patients in critical decisions about their health [26].

Our paper has limitations. We did a qualitative analysis of survey responses, which did not enable us to ask follow-up questions to further explore a theme. Analyzing written responses, without further clarification from respondents, could result in misclassification of certain statements. Though we sought to have a representative national response, our study was limited by having a low response rate from British Columbia and Quebec, and from some patient types (First Nations and the elderly). While we think that many of the views expressed in this report will be common across kidney care programs, the extent of the issue may vary based on geographic regions, health care systems and the availability of local resources. The information provided by this study could be supplemented by local questionnaires to best assess local needs, and inform local program changes.

## Conclusions

We identified four themes from this analysis of the views of patients’ on or nearing dialysis and their caregivers: gaining knowledge, maintaining quality of life, sustaining psychosocial wellbeing, and ensuring appropriate care. Importantly, respondents identified a variety of issues that could be addressed by both health systems and health care providers to improve their quality of life. These include the development of patient materials and resources, or sharing of existing resources across Canadian renal programs, along with adopting better communication strategies. Other concerns, such as the need for increased psychosocial and financial support, require consideration by health care funders.
